# 507 Capturing Growth and Change Through the Burn Camp Experience

**DOI:** 10.1093/jbcr/irac012.138

**Published:** 2022-03-23

**Authors:** Kerry Mikolaj, Brad Jackson, Trudy Boulter, Nichole M Schiffer

**Affiliations:** Children's Hospital Colorado, Westminster, Colorado; Children's Hospital Colorado, Aurora, Colorado; Children's Hospital Colorado, Denver, Colorado; Children’s Hospital Colorado, Denver, Colorado

## Abstract

**Introduction:**

The American Camping Association (ACA) has asked in their study titled, *Youth Development Outcomes of the Camp Experience: Evidence for Multidimensional Growth*, “In what ways do children change because of camp experiences?” Our burn camp program wanted to answer this same question about the medical specialty camp experience. Camp counselors and staff have been addressing this question in their camp summary reports for years, focusing on independence, social relationships, and adventure/risk taking. This project asked campers and caregivers/parents to rate their experiences of change in these key areas of growth and development.

**Methods:**

The ACA supported the development of the Camper Growth Index instrument. We selected items from this larger scale to address three areas of change in our burn camp population: Independence/Leadership, Adventure/Exploration, and Social Skills. Three to four items were selected for each area based on the strength of their factor scores in the original study, relevance to our medical specialty camp setting, and the growth we were already documenting in our counselor reports.

Campers ages 8–18 and their caregivers/parents rate items addressing these areas on a 4-point Likert scale (disagree a lot, disagree a little, agree a little, agree a lot). Sample questions are “I am good at doing things on my own”, “I like to talk to kids I don’t know yet”, and “My child likes to try new activities”. Items are phrased in positive and negative directions (reversed scored).

All 60 campers and caregivers received these questions prior to camp. The same questions will be sent 3 months post camp to assess change in these areas.

**Results:**

Preliminary findings from the pre-camp responses indicate positive evaluations in these three areas. We will be gathering post-camp responses from youth and caregivers / parents to assess change over the camp experience and return to home and school.

**Conclusions:**

Burn camps are a vital part of the burn rehabilitation and aftercare experience for young burn survivors and their families. Documenting the change we all believe happens at burn camp (and hear about anecdotally from our campers and families) remains an important task. The Camper Growth Index utilized by the broader ACA study allows us to assess the camper and caregiver/parent experience of this change.

